# Timescales of Human Hair Cortisol Dynamics

**DOI:** 10.1016/j.isci.2020.101501

**Published:** 2020-08-26

**Authors:** Lior Maimon, Tomer Milo, Rina S. Moyal, Avi Mayo, Tamar Danon, Anat Bren, Uri Alon

**Affiliations:** 1 Department Molecular Cell Biology, Weizmann Institute of Science, Rehovot 76100, Israel

**Keywords:** Biological Sciences, Human Metabolism, Chronobiology

## Abstract

Cortisol is a major human stress hormone, secreted within minutes of acute stress. Cortisol also has slower patterns of variation: a strong circadian rhythm and a seasonal rhythm. However, longitudinal cortisol dynamics in healthy individuals over timescales of months has rarely been studied. Here, we measured longitudinal cortisol in 55 healthy participants using 12 cm of hair, which provides a retrospective measurement over one year. Individuals showed (non-seasonal) fluctuations averaging about 22% around their baseline. Fourier analysis reveals dominant slow frequencies with periods of months to a year. These frequencies can be explained by a mathematical model of the hormonal cascade that controls cortisol, the HPA axis, when including the slow timescales of tissue turnover of the glands. Measuring these dynamics is important for understanding disorders in which cortisol secretion is impaired over months, such as mood disorders, and to test models of cortisol feedback control.

## Highlights

• Longitudinal cortisol over a year was measured using hair from 55 healthy individuals
• A method was developed to adjust for decline along the length of the hair
• Cortisol showed non-seasonal fluctuations with periods of months to a year
• These fluctuations can be explained by turnover time of cortisol-related glands

## Graphical Abstract

**Figure:**
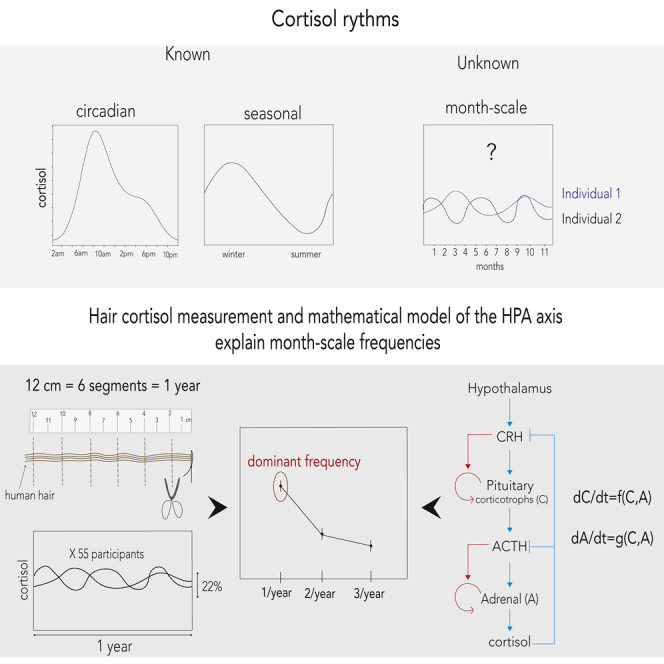

## Introduction

Cortisol is a major stress hormone in humans. It is secreted in response to psychological and physiological stress, under control of a cascade of hormones called the HPA (hypothalamus-pituitary-adrenal) axis. Cortisol has receptors in most cell types and exerts widespread actions that help the organism to prepare for stressors and to cope with them (Buckingham, 2009; McEwen, 1998; Sapolsky et al., 2000). Cortisol dysregulation is implicated in physiological and psychological pathologies, such as mood disorders, including depression (Daban et al., 2005; Ehlert et al., 2001; Nemeroff et al., 1992; Pearson Murphy, 1991; Sriram et al., 2012; Watson and Mackin, 2006; Wingenfeld and Wolf, 2015).

**Figure 1:**
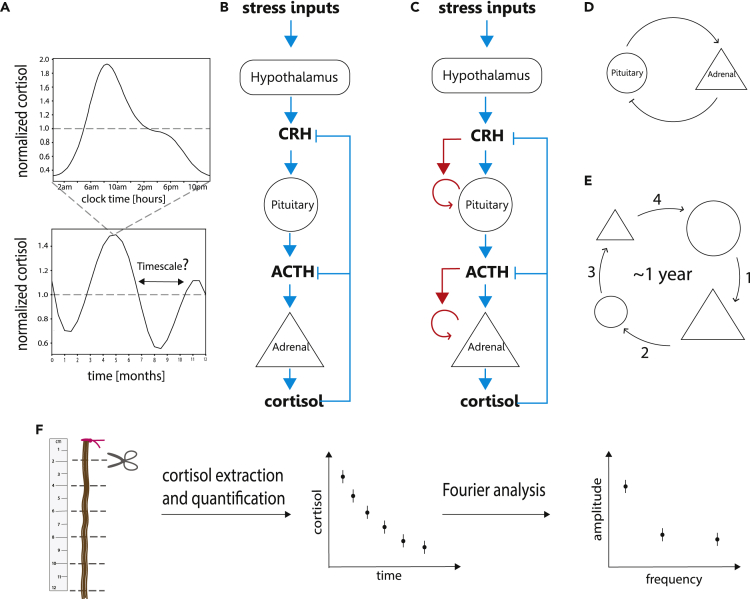
Cortisol Secretion Is Governed by the HPA Axis (A) Top panel: cortisol shows circadian changes over the day, adapted from Chan and Debono, (2010). Bottom panel: varying stress inputs can cause fluctuations over months, whose timescale is the subject of this study. (B) Classic model of the HPA axis in which stress inputs cause corticotropin-releasing hormone (CRH) secretion from the hypothalamus, causing the pituitary corticotrophs to secrete adrenocorticoptropic hormone (ACTH), which in turn causes cells in the adrenal cortex to secrete cortisol. Cortisol inhibits the secretion of the upstream hormones. (C) The Karin et al. model, which includes the effect of hormones on gland proliferation (red arrows), which introduces the slow timescale of tissue turnover (weeks-months). (D) The adrenal cortex and pituitary corticotrophs effectively form a negative feedback loop when considering the slow timescale of months. (E) An increase in pituitary corticotroph cells' total mass leads to increased ACTH secretion, which increases adrenal cortex mass (1). This leads to an increase in cortisol, which inhibits ACTH, causing a reduction in corticotroph cells (2). This feedback loop with cell turnover times of few weeks has a resonance frequency with an overall timescale of a year. (F) Schematic overview of this study: longitudinal hair cortisol over 1 year is analyzed using Fourier transform to detect frequencies of fluctuations.

Cortisol has dynamics on several timescales (Figure 1A). It shows ultradian pulses throughout the day that last about 60–90 min (Young et al., 2004). Pulse amplitude is largest in the morning, forming a sizable circadian pattern (Walker et al., 2010). Cortisol also has a seasonal rhythm, which peaks in late winter (Hadlow et al., 2018; Miller et al., 2016; Tendler et al., 2020). The amplitude of this seasonal rhythm is a few percent in temperate clines and rises to tens of percents at high latitudes. Detecting this seasonal rhythm requires averaging over many individuals. On the timescale of decades, cortisol generally decreases at old age on average (Sharma et al., 1989).

Overlaid on top of these daily and seasonal patterns is the response of cortisol to the stressors that occur over time. Thus, one expects cortisol dynamics to fluctuate over weeks, months, and years in each individual. However, the nature of these slow fluctuations and their typical timescale have not been quantified.

Understanding fluctuations on a timescale of months is important to better understand the feedback loops in the HPA axis. Cortisol inhibits its upstream hormones (Figure 1B) in a classic feedback loop that acts on the timescale of minutes to hours (Andersen et al., 2013; Ronald De Kloet et al., 1998). This feedback can explain ultradian rhythms on the scale of hours (Walker et al., 2010). A recent model by Karin et al., 2020 points to an additional feedback loop, which acts over months. In this feedback loop, the functional mass of the glands in the HPA axis changes over time, under the control of the hormones that act as growth factors (Bicknell et al., 2001; Gertz et al., 1987; Horvath et al., 1999; Kataoka et al., 1996; Nolan et al., 1998; Westlund et al., 1985) (Figure 1C). This effectively forms a feedback loop between two glands in the HPA axis, the adrenal cortex cells and the pituitary corticotrophs (Figure 1D). This feedback loop is predicted to have a typical timescale of about a year, due to the slow timescale of tissue turnover (Figure 1E). Intuitively, this model predicts that stressors over time would stimulate the natural period of this feedback loop, leading to noisy fluctuations of cortisol with a timescale of about a year (added on top of the much smaller seasonal pattern).

Understanding cortisol fluctuations in healthy individuals can also set a baseline to compare with the situation in mood disorders that play out over months, such as depression and bipolar disorder (Belvederi et al., 2016). Future studies of cortisol dynamics in individuals with mood disorders would benefit from a solid understanding of the control group, namely, cortisol fluctuations in healthy individuals.

Here we explore the dynamics of cortisol in healthy participants using longitudinal measurements over 1 year. We use hair cortisol, a measure that allows retrospective quantification (D'Anna-Hernandez et al., 2011; Davenport et al., 2006; Mayer et al., 2019). Cortisol passively lies in hair. Each centimeter of hair corresponds to about 1 month of growth, and thus analyzing hair allows measurement of cortisol levels averaged over long times (D'Anna-Hernandez et al., 2011; Smy et al., 2016). We developed a procedure to adjust for the decay of cortisol over 12-cm hair samples and use Fourier analysis to quantify the contribution of different frequencies in the signal. We find fluctuations with a standard deviation of about 22% around each individual's baseline and a dominant, non-seasonal frequency component of 1 year. We show that the gland-mass feedback loop model can explain these frequencies.

## Results

### Longitudinal Cortisol Fluctuates around Its Baseline

**Figure 2:**
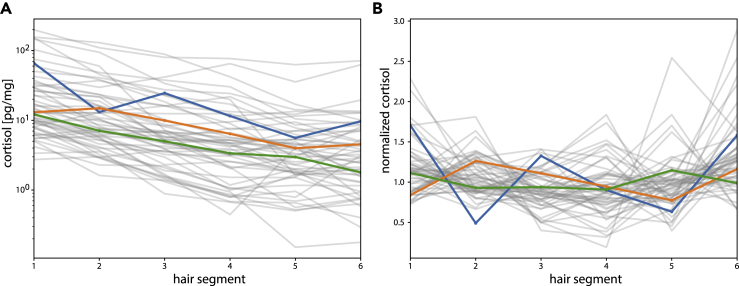
Longitudinal Cortisol Measurements from Human Hair (A) Cortisol time series from 55 participants. Each time series has 6 points corresponding to six 2-cm segments of hair, representing about 1 year of growth. (B) Normalized cortisol for the same participants, after correction for decline along the hair. Highlighted in color are three examples of individual cortisol time series.

We collected 12 cm of hair from healthy participants and assayed cortisol using ELISA in 2-cm segments. This provided a longitudinal time-series of six time points over about a year of growth (Figure 2A).

We corrected each time series for the decline of cortisol in distal segments by fitting an exponential decay model to each participant's cortisol measurements (see Methods). The mean decay coefficient was $\bar{\alpha }\;=\;2.2\;\pm \;0.2\;\text{[}{\mathrm{year}}^{-1}\text{]}$ (standard error of the mean, SEM). The exponential fit allowed estimate of the baseline cortisol for each individual. The baseline varied about 30-fold between individuals, as can be seen in Figure 2A.

After normalizing by the decline, the fluctuations around each individual baseline can be seen in Figure 2B.

Normalized cortisol showed fluctuations around the mean with an average coefficient of variance (CV) of 28%. When accounting for experimental noise with CV = 14% (see Methods), one obtains fluctuations with CV = 24%.

### Longitudinal Hair Cortisol Shows a Dominant 1 Year^−1^ Frequency Component

To explore the frequencies underlying these fluctuations, we used Fourier analysis, which quantifies the contributions of different frequencies to the signal. Six segments allow three frequencies to be detected: 1 [*year*^*−*1^], 2 [*year*^−1^], and 3 [*year*^−1^], representing periods of a year, 6 months, and 4 months, respectively.

**Figure 3:**
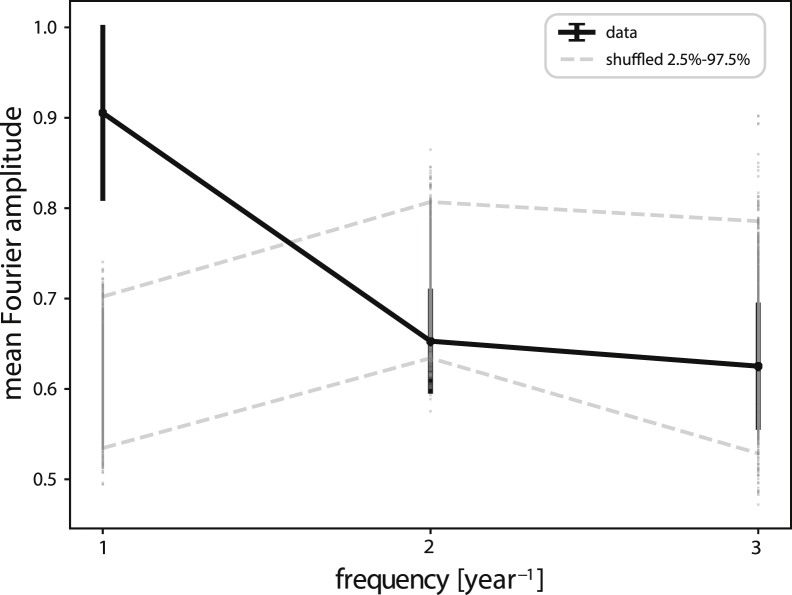
Hair Cortisol Shows Fluctuations with a Dominant Period of 1 Year Fourier amplitudes averaged over participants quantify the contribution of each frequency component (1[*year*^−1^], 2[*year*^−1^], and 3[*year*^−1^]) to the cortisol signal (black dots). Error bars (SEM) were calculated by bootstrapping the participants. Shuffled control is shown as gray dots (1,000 repeats), with 97.5% and 2.5% confidence intervals shown in dashed gray lines. The amplitude of the 1 [*year*^−1^] frequency is significantly higher than shuffled control, p = 0.004.

We averaged the Fourier amplitudes over the participants. The highest mean amplitude was obtained at the slowest frequency, 1 [*year*^−1^] (Figure 3, black dots). This amplitude was about 1.45 ± 0.17 times higher than the amplitude at the highest frequency, 3 [*year*^−1^].

To test the statistical significance of this observation, we compared the Fourier spectrum to a null model constructed by shuffled data and subjected to the same correction for cortisol decline (Methods, Figure 3, gray dots). The amplitude of the 1 [*year*^−1^] frequency was significantly higher than shuffled control with a large effect size (p = 0.004, Cohens d = 3.8, non-parametric p = 0.002, Cliff's delta = 0.99).

Note that the null model has lower amplitudes in the 1 [*year*^−1^] frequency compared with the other frequencies. This decrease in the slowest frequency is due to correction for the cortisol decline along the hair: the subtraction (in log-transformed variables) of an exponential fit from the raw signals removes a major portion of the slowest frequency component from the normalized signals. This is generally true also for random data, such as those generated from a normal distribution. The experimental data show high amplitude at low frequency (1 [*year*^−1^]) despite this effect.

We also conducted a non-parametric correction for cortisol decline (see Methods Supplemental Information, section S4), which gave the same conclusion of a dominant 1 [*year*^−1^] frequency component in cortisol spectrum.

We also corrected for seasonality—the effect of the month of the year on cortisol—by fitting the decline-corrected cortisol to a cosinor model. Cortisol showed a seasonality effect of about 15%. When corrected for seasonality (Supplemental Information, section S3), the CV of the decline-corrected fluctuations dropped from 24% to 22%. The *year*^−1^ frequency remains the dominant frequency. This indicates that the slow cortisol variations in each individual go beyond a seasonal effect.

#### Model of the HPA Axis with Gland Mass Dynamics can Explain the Year-scale Fluctuations

We asked what biological processes might underlie the observed frequency spectrum of hair cortisol, with large amplitude at *year*^−1^ frequency. One possibility is that stressor inputs to the HPA axis have dominant low-frequency components. For example, due to life events, some periods of several months may be more stressful than others (Dettenborn et al., 2010; Rothman, 1972; Staufenbiel et al., 2013), contributing to the observed fluctuations (beyond the variation with season).

Here we consider in detail an alternative explanation, in which the interactions within the HPA axis contribute to the low-frequency cortisol fluctuations. We thus ask whether stressor inputs randomly distributed over time can generate cortisol fluctuations with typical fluctuations of a year.

**Figure 4:**
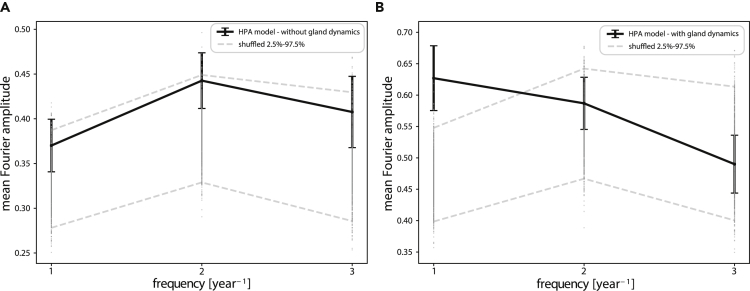
HPA Model with Gland Mass Dynamics Is Sufficient to Explain the Year-Scale Fluctuations in Cortisol (A) Textbook HPA model includes a cascade of hormones with negative feedback of cortisol on its upstream hormones (Figure 1B). The Fourier spectrum in response to white noise input is similar to the null model. (B) Karin et al. model with gland mass dynamics (Figure 1C) shows a Fourier spectrum with a dominant [*year*^−1^] frequency component. The black dots are the mean Fourier amplitude with SEM error bars. Dashed lines: 2.5% and 97.5% confidence intervals of a shuffled data control. Both simulations and null models were subjected to the decline correction.

We simulated and compared two models of the HPA axis. First is the classic HPA model (Figure 1B), in which the only feedback mechanism is inhibition by cortisol to upstream hormones. This model has timescales given by the hormone half-lives, namely, hours. Simulating this model with white-noise stress input results in a Fourier spectrum without dominant *year*^−1^ frequency. Instead, all frequencies are found with approximately the same amplitude. Applying the decline correction to the simulated data, as done to the experimental data, results in a spectrum that is indistinguishable from the null model (Figure 4A).

In contrast, we simulated the model of Karin et al. (Figure 1C), which includes the changes of gland masses with typical timescale of months. Using the same input signals, this model provides a dominant 1[*year*^−1^] frequency component (Figure 4B). The period of 1 year arises from the tissue turnover times, which are on the order of a few weeks, due to the 2π term in the equation period = 2π/frequency. The only model parameters that affect the timescales of months-years are the two tissue turnover times. All other model parameters are of the fast timescale of hours, such as hormone turnover times and secretion rates, and do not contribute to the dynamics on this slow timescale. This can be shown analytically using the Laplace transform on linearized model equations (Supplemental Information, section S6), as well as by a detailed sensitivity analysis using simulations (Supplemental Information, section S5).

We find that the turnover times of the two tissues in the model, corticotrophs and cortisol-secreting cells in the adrenal cortex, can range from days to months to obtain a similar shape to the observed Fourier spectrum. Thus, we conclude that the model is sufficient to explain the timescales of the measured cortisol fluctuations.

## Discussion

We analyzed longitudinal hair cortisol in healthy participants. Cortisol fluctuated by about 22% around the baseline, with variations that have a dominant low-frequency component with a period of 1 year. This variation goes beyond the effect of seasonality. The slow variations are consistent with a recent model of the HPA axis, which can explain low frequencies by the hormonal regulation of the functional mass of the cells that secrete adrenocorticoptropic hormone and cortisol, namely, pituitary corticotroph cells and adrenal cortex cells. The timescale of months to years arises due to the slow turnover time of the tissues.

Hair has advantages for longitudinal cortisol profiling on the timescale of months, due to its low invasiveness, ease of acquisition, convenient storage, and the ability of hair to record cortisol from the past. A technical advance in this study was to correct for the decay in cortisol distally along the hair. This decay was reported in several previous studies and has led many researchers to use only the first few centimeters of hair closest to the scalp (Kirschbaum et al., 2009; Mayer et al., 2019; Schonblum et al., 2018). Here we corrected the decline by fitting an exponential decay to each time series and compared this to shuffled controls. This allowed us to estimate the extent and significance of the Fourier amplitudes over a year of dynamics.

One limitation of this study is that 12 cm of hair does not allow rhythms slower than 1 year to be detected. Indeed, the HPA model suggests that such slower rhythms are expected. As cortisol in most samples decayed close to background detection levels after 12 cm, a future longitudinal study with multiple hair samples is needed to estimate the amplitude of slower Fourier frequencies.

The present agreement with the Karin et al. HPA model adds to a picture in which the functional masses of the cells in the HPA axis are important variables. These masses are not considered in standard models of the HPA axis. Karin et al. showed that such mass changes are sufficient to explain HPA dysregulation after prolonged stress. (Tendler et al., 2020) proposed that the same model can explain seasonal entrainment of hormones and their seasonal peaks and troughs. Here, we propose that the mass changes provide a “memory” to the HPA axis, which can integrate over the fast timescale fluctuations of stress inputs to generate fluctuations that last on the timescale of a year. Future work can further test this model by measuring gland masses over time and correlating them with hormonal measurements.

The HPA model studied here is not the only possible explanation for the slow fluctuations with periods of months to years. These fluctuations may also arise due to slow timescales in the distribution of stressors over time. Such slow timescales arise in many human activity patterns, which have long-tailed distributions of intervals between events (Vázquez et al., 2006). Investigating the role of the temporal stressor distribution requires following stressors over time and may be the focus of future work. Additional effects can introduce long timescales, including the effects of epigenetic regulation in the HPA axis.

The present study can serve as a baseline for future exploration of stress-related pathologies. For example, mood disorders display HPA dysregulation, but the precise dynamics of this dysregulation remains to be clarified in conditions such as bipolar disorder. Hair offers a rare opportunity to look retrospectively from the time of first diagnosis, showing the dynamical prodrome to disease onset. Cortisol dynamics can be used to test models of pathology, and to provide a detailed diagnostic for HPA axis function.

In the long term, one may envision using hair cortisol longitudinal dynamics as a basis for stabilizers of psychological diseases that involve dysregulation of the HPA axis on the scale of months (other HPA syndromes such as addisonian crisis can have much faster timescales and cannot rely on hair measurements). In this paradigm, the goal is to return dysregulated HPA function back to baseline using a feedback-control approach. One measures cortisol, simulates a model of the axis, and determines the optimal dose of HPA agonists or antagonists to take in the next time period (e.g., a month) to return dysregulated HPA function back to baseline (Ben-Zvi et al., 2009). Following the interventions, hair measurements can be used to test if the desired state was reached.

### Limitations of the Study

This study involved a sample of only 55 individuals from one country. Future work can enlarge sample size and sample additional populations, which is important given the large person-to-person variability in cortisol. Use of other methods to measure cortisol, such as mass spectrometry, can test the validity of the results. Use of multiple short hair samples from the same individual can extend the study period beyond 12 months and test the validity of the decline correction.

### Resource Availability

#### Lead Contact

Further information and requests for resources should be directed to and will be fulfilled by the Lead Contact, Uri Alon (uri.alon@weizmann.ac.il).

#### Materials Availability

This study did not generate new unique reagents.

#### Data and Code Availability

The data and code generated during this study are available at https://github.com/tomermilo/hair-cortisol.

## Methods

All methods can be found in the accompanying Transparent Methods supplemental file.

## Supplemental Information

Document S1. Transparent Methods, Figures S1–S5, and Table S1

## Footnotes

Supplemental Information can be found online at https://doi.org/10.1016/j.isci.2020.101501.
