# Supplementary material for: Timescales of Human Hair Cortisol Dynamics
Source: iScience. 2020 Aug 26;23(9):101501. doi: 10.1016/j.isci.2020.101501 (PMC7490542; doi:10.1016/j.isci.2020.101501)
Supplement: Document S1. Transparent Methods, Figures S1–S5, and Table S1 [file mmc1.pdf]

iScience, Volume *23*

Supplemental Information

Timescales of Human

Hair Cortisol Dynamics

Lior Maimon, Tomer Milo, Rina S. Moyal, Avi Mayo, Tamar Danon, Anat Bren, and Uri Alon

Supplemental Information for "Timescales of human hair cortisol dynamics over one year"

Transparent Methods

Ethics statement

The study protocol was approved by the Review Board of the Weizmann Institute of Science (study code: 706-1). Written informed consent was obtained from all participants.

Participants

Healthy participants (N=59, females=45, average age=27±6) were recruited during January-December of 2019 through social media. Inclusion criteria: participants older than 18 years, with at least 12 centimeters of natural hair with no cosmetic treatment such as dying or perming (Cooper, 2015). Exclusion criteria: diagnosis of a mental, psychological or endocrine disorder, consumption of steroids, psychiatric drugs or other drugs that might affect the endocrine system during the year prior to participation, and pregnancy in the year before participating. Oral contraceptives were allowed as long as there was no change in prescription during the year prior to participation. Each subject was asked to complete a personal information questionnaire prior to the collection of a hair sample. The study was anonymous; each hair sample received a serial number. Male height was 179±6 cm, weight 73±11 kg and BMI (body mass index) 22±3; Female height was 164±6 cm, weight 61±9 kg and BMI 23±3 (all values are mean±STD). 26% of the females reported taking oral contraceptives.

Hair Cortisol Measurements

A lock of hair (a pencil-width group of about 100 hair strands) from the vertex posterior area (Cooper, 2015; Sauvé et al., 2007) of the head was tied with a thread and cut with fine scissors as close to the scalp as possible. We estimate that hair is cut at 0.8±0.1 cm from the scalp, in agreement with Cooper et al. (2015). Cut samples were masking-taped at their distal end to a piece of aluminum foil; the tied thread marked the proximal end. Samples were kept in the laboratory at room temperature before analysis.

We adapted a protocol by Schonblum et al. (2018) for the extraction and measurement of hair cortisol. The first 12 centimeters of each hair sample, starting from the proximal end, were segmented to six 2 cm segments (segments of 1 cm dropped below the detection threshold too often and thus 2 cm segments were used). The segments were placed in vials (Fisherbrand, 21x70 mm) and washed twice with 5 ml isopropanol while

mixing on an orbital rotator for 3 minutes. Isopropanol was then decanted and the open vials were left in a chemical hood to dry overnight. Then, 2 ml of methanol was added to each vial, sonicated for 60 min, and incubated overnight (approximately 20 hours) at 50°C while shaking. The following day, all the methanol was transferred to 2 ml Eppendorf tubes and centrifuged for 10 minutes at 4°C. Methanol (1.5 ml) from each tube was transferred to a glass vial (Falcon, 12x75 mm) and evaporated under a stream of nitrogen at 45 °C. Samples were reconstituted in 10% methanol and 90% assay buffer provided by the kit manufacturer and cortisol was quantified using competitive Enzyme-Linked Immunosorbent Assays (ELISA; Salimetrics Europe, Newmarket, cat.no1-3002-5 for cortisol, UK). Reported antibody cross-reactivity was 19.2% with dexamethasone, and less than 1% with 15 other tested steroids. Linearity was observed between 30-70 mg of hair, hence we used 30-70 mg of hair to measure cortisol. The assay detection threshold was 112 pg as specified by the manufacturer. All 6 segments from the 12 cm sample of hair from each participant were analyzed in the same batch of washing, sonication, extraction and ELISA plate. To control for inter-assay variation, we generated a standard curve for each plate, consisting of 6 known concentrations of cortisol supplied by the kit manufacturer assayed in 6 wells. To estimate the inter-batch variation, we assayed multiple standard hair samples. Each standard sample was taken from a large, well-mixed, sample of hair collected from a single individual. The coefficient of variation (CV=STD/mean) of 13 standard samples measured on 2 different day was 14%.

We included in this study the 55 participants (32 females) with all 6 cortisol measurements above detection threshold. Mean cortisol levels did not show a significant correlation with age (spearman r=0.19, p=0.2), weight (spearman r=0.01, p=0.9), height (spearman r=0.06, p=0.7), and BMI (spearman r=0.03, p=0.8). Mean cortisol levels did not significantly differ between the sex groups (Mann Whitney U test, p=0.22) or by taking oral contraceptives (Mann Whitney U test, p=0.35).

Analysis of cortisol time series

Each hair sample provided six 2 cm segments. The average rate of scalp hair growth in humans is approximately 1 cm/month, with a reported range of 0.6 to 1.5 cm a month (Cooper, 2015); we thus assumed that each 2 cm segment represents two months of growth and contains cortisol that accumulated during that period. Due to factors including hair washing (Hamel et al., 2011), cortisol levels in hair decline as a function of distance from the scalp (Gao et al., 2010; Kirschbaum et al., 2009; Steudte et al., 2011). Studies on glycosylated proteins in hair showed a similar decline that was well-described as exponential with time (Nissimov et al., 2007). To account for the decline, we performed a linear regression on the log of the measurements, equivalent to assuming an exponential decline of $A_j exp(-\alpha_j t_i)$ for individual j, where t_i is the time corresponding to segment $i = 1...6$, assuming hair growth rate of $1\ cm/month$. We constrained the slope to be negative

($\alpha_j > 0$). Thus, if $\hat{c}_{ij}$ is the raw cortisol measurement, we define $z_{ij} = log(\hat{c}_{ij})$, then use linear regression on z_{ij} to define the decline as $d_{ij} = log(A_j) - \alpha_j t_i$, and subtract this from z_{ij} to obtain the normalized log cortisol in segment i for person j, $y_{ij} = log(c_{ij}) = z_{ij} - d_{ij}$. Fourier analysis was computed on $c_{ij} = exp(y_{ij})$ using the dFFT function of python v3.7.4, numpy v1.16.5.

Fourier analysis is a widely-used method to decompose a time-varying signal into its constituent frequency components. It provides a breakdown of the signal into a sum of sine waves of different frequencies. Each sine wave has an amplitude and a phase. The higher the amplitude at a given frequency, the higher the contribution of that frequency to the signal.

The number of different frequencies provided by this analysis equals half of the number of time-points in the signal. For a signal with six time-points, three frequencies are available: the lowest frequency corresponds to a period equal to the total duration D of the 6 measurements, and the two other frequencies correspond to periods that are 1/2 and 1/3 of D. In the present case, D=1 year, and the frequencies correspond to sine waves with periods of 1 year, 6 months and 4 months.

We also estimated the effect of the month of the year on cortisol measurements. For this purpose, we averaged the decline-corrected c_{ij} according to the calendar month corresponding to the middle of the segment, taking into account an offset of 3mm inside the scalp and 8mm outside the scalp at the point of hair cutting, and a growth rate of 1cm/month. We then fit the resulting average, denoted $C(t)$ where t is the month of the year, to a cosinor model $Acos(\omega t + \phi)$ with $\omega = \frac{2\pi}{12}\frac{rad}{month}$. To correct for month of the year, we then normalized c_{ij} by the best-fit cosinor model using the month of the year for each mid-segment (see SI, S3).

To estimate significance, we compared our results with the Fourier analysis of a shuffled control. We shuffled the segments $i = 1...6$ within each participant's normalized data c_{ij}. This yielded shuffled data, s_{ij}. The shuffling keeps each participant's normalized cortisol distribution but breaks temporal correlations. We then added the best-fit decline of that participant, to get simulated raw log data, $z'_{ij} = \log(s_{ij}) + d_{ij}$. We then repeated the analysis by fitting the decline with a new regression (which yields a slightly different decline, d'_{ij}, due to the data shuffling), subtracted the decline d'_{ij} to obtain $y'_{ij} = z'_{ij} - d'_{ij}$, and performed the same Fourier analysis on $exp(y'_{ij})$. This controls for the fact that the decline correction affects the long-wavelength components of the data. We repeated this procedure 1,000 times in order to estimate statistical significance (See SI, S2 for details).

We also performed a second, non-parametric analysis. We assumed that the decline of cortisol is monotonic with distance from the proximal segment. We therefore performed a rank regression on $\hat{c}_{ij}$ versus segment number ($i = 1\ldots6$). We then subtracted the rank regression from the rank of $\hat{c}_{ij}$, to obtain the rank residuals r_{ij}. Finally, we performed a Fourier analysis on r_{ij}. As a shuffled control, we shuffled r_{ij} within each participant j, added the rank regression for that participant, rank-regressed again, and performed Fourier analysis on the residuals. The results are qualitatively similar to the parametric test and are shown in the SI, S4.

HPA model

We employ a recently developed model for the HPA axis, which incorporates the effects of the hormones on the total functional mass of hormone-secreting cells (Karin et al., 2020; Tendler et al., 2020). The concentrations of the hormones CRH, ACTH and cortisol are denoted x_1, x_2 and x_3. The input to the hypothalamus, which describes the combined impact on CRH secretion due to physiological, circadian, and psychological stressors is denoted u. The total functional mass of pituitary corticotroph cells that secrete ACTH is C, and that of the adrenal cortex cells that secrete cortisol is A. The secretion of CRH due to input u is as follows:

(1) $\frac{dx_1}{dt} = b_1\, u f(x_3) - a_1 x_1$

Where b_1 is the secretion parameter of CRH, and a_1 is CRH removal rate. $f(x_3)$ describes the feedback by cortisol, due to the mineralocorticoid and glucocorticoid receptors (MR and GR, respectively) in the hypothalamus, $f(x_3) = MR(x_3)\, GR(x_3)$. Since the high-affinity receptor MR is usually bound by cortisol at physiological levels we use an approximation to the Michaelis–Menten binding kinetics; GR is a cooperative (n) receptor that binds cortisol with lower affinity, K_{GR}:

(2) $MR(x_3) = \frac{1}{x_3}$

(3) $GR(x_3) = \frac{1}{1+(x_3/K_{GR})^n}$

The dynamics of ACTH are as follows:

(4) $\frac{dx_2}{dt} = b_2 x_1 C g(x_3) - a_2 x_2$

While b_2 is the secretion parameter per unit corticotroph functional mass. The parameter b_2 includes per-cell effects with signaling pathways such as CRH receptor numbers per cell, neuronal inputs and cytokine inputs that affect corticotrophs. $g(x_3)$ is the feedback from cortisol due to the GR receptors in the pituitary, $g(x_3) = GR(x_3)$.

The dynamics of cortisol are as follows:

$$(5)\ \frac{dx_3}{dt} = b_3 x_2 A - a_3 x_3$$

While b_3 includes all per-cell effects on cortisol secretion rates, and a_3 is cortisol removal rate. To this classical model, Karin et al. added the effects of the hormones on the total functional mass $C(t)$ and $A(t)$, which are important for the present study. The mass changes can be due to cell division (hyperplasia) or cell growth (hypertrophy); the precise mechanism is not important for the present analysis. The main growth factor for corticotrophs is CRH, so that proliferation rate is $b_C x_1$ and removal rate is a_C, resulting in the following:

$$(6)\ \frac{dC}{dt} = C(b_C x_1 - a_C)$$

Note that C occurs in both proliferation and removal terms, because differentiated corticotrophs divide to produce new corticotrophs (Gulyás et al., 1991) (with additional supply, not considered here, from pituitary stem cells (Andy Levy, 2007; Nakane et al., 1977)). The main growth factor for cortisol-secreting cells in the adrenal cortex is ACTH and therefore:

$$(7)\ \frac{dA}{dt} = A(b_A x_2 - a_A)$$

The timescales for the change in mass are governed by the removal rates a_A and a_C, which are experimentally found to be on the scale of weeks in model organisms (A Levy, 2002; Nolan et al., 1998; Swann, 1940; WESTLUND et al., 1985). An analytical solution of the steady state stability found a spiral fixed point with a period on the order of a year (Tendler et al., 2020). The parameter values used in the present simulations are given in table 1 (SI, S1). In a version of the model without cell mass dynamics, we used constant $A(t) = C(t) = 1$ and omitted equations 6 and 7. We numerically solved the model using python's solver, 'odeint' of scipy v1.3.1 (Virtanen et al., 2020) The input was piecewise-constant u in every 2-month time period, with a value of u drawn from a lognormal distribution with a mean of 1 and a standard deviation (STD) of 0.65, determined to give cortisol STD similar to measurements. We simulated four years of dynamics, and took the last year for analysis, in order to avoid transients due to initial conditions. We simulated individual participant data by multiplying x_3 by the exponential decline fit for that participant and averaging this over six consecutive 2-month periods. This generated a simulated dataset with the same number of participants as the experimental data. We then performed the same analysis as for the experimental cortisol measurements. To test the significance of the model results, we repeated this procedure 1,000 times with new simulations.

To complement the empirical analysis with simulations we performed an analytical spectral analysis on the linearized model equations, using Bode plots (SI, S6).

S1. Table of reference parameter values, related to Figure 4

Parameter	Value	Reference
b_1	0.17 [1/min]	(Andersen et al., 2013)
b_2	0.035 [1/min]	(Andersen et al., 2013)
b_3	0.0091 [1/min]	(Andersen et al., 2013)
b_P	1/30 [1/day]	(Nolan et al., 1998)
b_A	1/30 [1/day]	(Kataoka et al., 1996)
K_{GR}	4	(Karin et al., 2020)
n	3	(Andersen et al., 2013)

S2. Statistical tests for significance of Fourier amplitudes

We tested the significance of the mean Fourier amplitude at $1[year^{-1}]$ compared to a shuffled control in which the 6 time-points of each individual are shuffled. For this purpose, we generated 1000 bootstrapped datasets in which we chose from the 55 participants with returns. For each bootstrapped dataset we computed the mean Fourier amplitude (A) at $1[year^{-1}]$. This results in a distribution $P_{bootstrap}(A)$. We then shuffled the time points of each participant and generated 1000 shuffled datasets. We computed the mean Fourier amplitude at $1[year^{-1}]$ for each shuffled dataset, to obtain $P_{shuffled}(A)$. We find that both distributions are very close to Normal, as expected for distributions of means. We then computed significance in two different ways, parametric and non-parametric. The parametric test used the normality of the distribution to calculate the weighted p value

and effect size (Cohens d) analytically. The non-parametric calculation asked how often $P_{shuffled}(A)$ exceeds $P_{bootstrap}(A)$. The effect size was calculated using Cliff's delta (whose range is [-1,1]).

S3. Correction for seasonality shows a dominant $\boldsymbol{year^{-1}}$ frequency

We also analyzed the data according to calendar months, using a cosinor analysis. Hair cortisol showed a seasonal amplitude of 15%±3 with a peak at May-June. We correct for seasonality by dividing the decline-corrected cortisol values for each segment by the cosinor model for the relevant months (Methods), and repeated the Fourier analysis.

Fourier analysis shows that the lowest frequency of 1 $year^{-1}$ remains the dominant frequency (Figure S1). It exceeds shuffled control significantly (p=0.01, Cohen's D=3.2, non-parametric p=0.01, Cliff's delta=0.98). We conclude that hair cortisol shows variations on the scale of a year that go beyond seasonality.

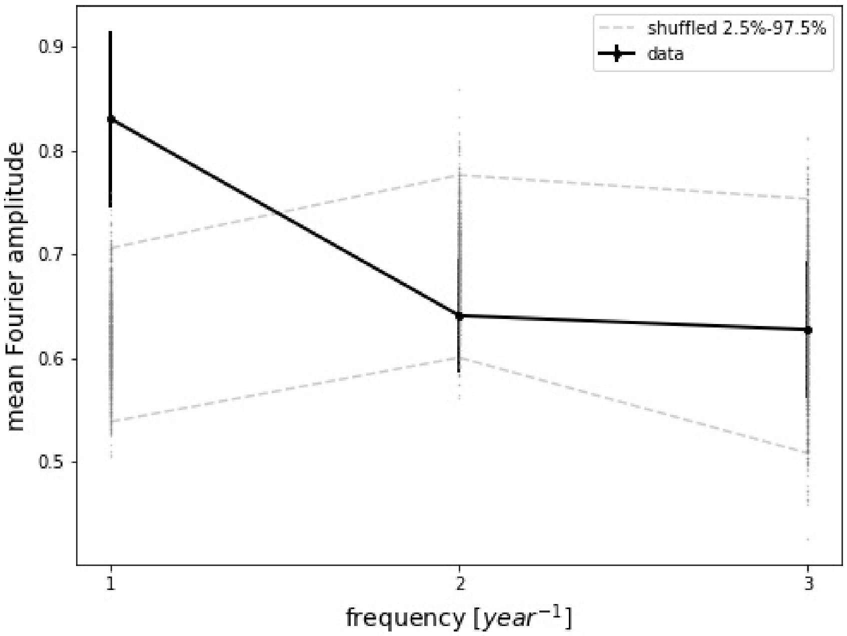

Figure S1. Hair cortisol shows fluctuations with a dominant period of 1 year, after correcting for seasonality, related to Figure 3. Fourier amplitudes averaged over participants quantify the contribution of each frequency component ($1[year^{-1}]$, $2[year^{-1}]$ and $3[year^{-1}]$) to the cortisol signal (black dots). Error bars (SEM) were calculated by bootstrapping the participants. Shuffled control is shown as gray dots (1,000 repeats), with 97.5% and 2.5% confidence intervals shown in dashed gray lines.

The present finding of a May-June peak of hair cortisol is not consistent with most previous cortisol seasonality studies that identify peak cortisol (acrophase) in winter (Hadlow et al., 2018; Persson et al., 2008; Tendler et al., 2020), including a study on hair cortisol on 3,507 British civil servants (Abell et al., 2016). There is one exception of a large study with a peak phase in summer - a study from Netherlands on 1,768 children (age 10-12) (Rosmalen et al., 2005). The reason for the discrepancy of the present peak season (acrophase) with most previous studies is not clear.

S4. Non-parametric correction for cortisol decline shows dominant $\boldsymbol{year^{-1}}$ frequency as well

We made a non-parametric correction for the cortisol decline along the hair by rank regression (see Methods). Despite the large loss of information due to using ranks, the $1\ year^{-1}$ frequency showed a trend of being higher than shuffled control (p=0.06, Cohen's d=2.2; nonparametric p=0.06, Cliff's delta=0.88) (Figure S2). The highest mean amplitude was obtained at the slowest frequency, $1\ year^{-1}$, this amplitude was about 1.3±0.2 times higher than the amplitude of the highest frequency, $3\ year^{-1}$.

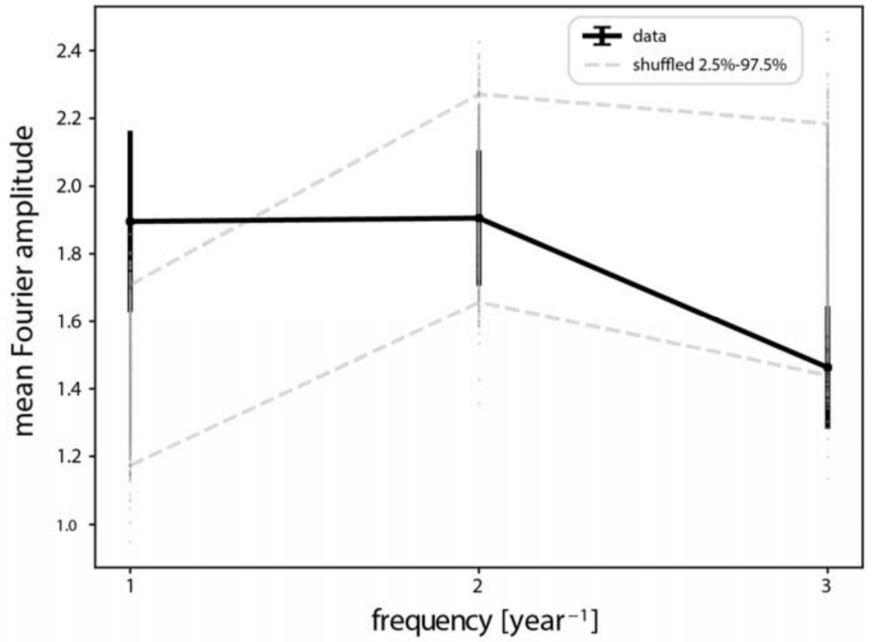

Figure S2. Rank-normalized hair cortisol shows fluctuations with a dominant year^{-1} frequency, related to figure 3. Mean Fourier amplitudes of hair cortisol data, which was normalized by rank regression (black dots with 1 STD error bars obtained by bootstrapping). Shuffled control is shown as gray dots (1,000 repeats), with 97.5% and 2.5% confidence interval shown in dashed gray lines.

S5. Sensitivity analysis of HPA model simulations

We tested the sensitivity of the main conclusion of the simulation analysis to the model parameters. We varied each of the five model parameters around its reference value (table S1) by a factor of up to $2^{10} = 1024$-fold. For each case, we repeated the simulation of Figure 4. We computed the ratio R between the $1\ year^{-1}$ and $3\ year^{-1}$ amplitudes. Figure S3 shows the percent change in R as a function of the fold-change in each parameter. The slow-timescale parameters for the tissue turnover processes, b_A and b_P, match the observed ratio within its experimental error (gray region) over an approximately 8-fold range around their reference values. The fast timescale parameters, that describe hormone production and removal, have minor effects on R across the entire 1024-fold range (inset). We conclude that the dominance of the $1\ year^{-1}$ frequency is insensitive to the model parameters.

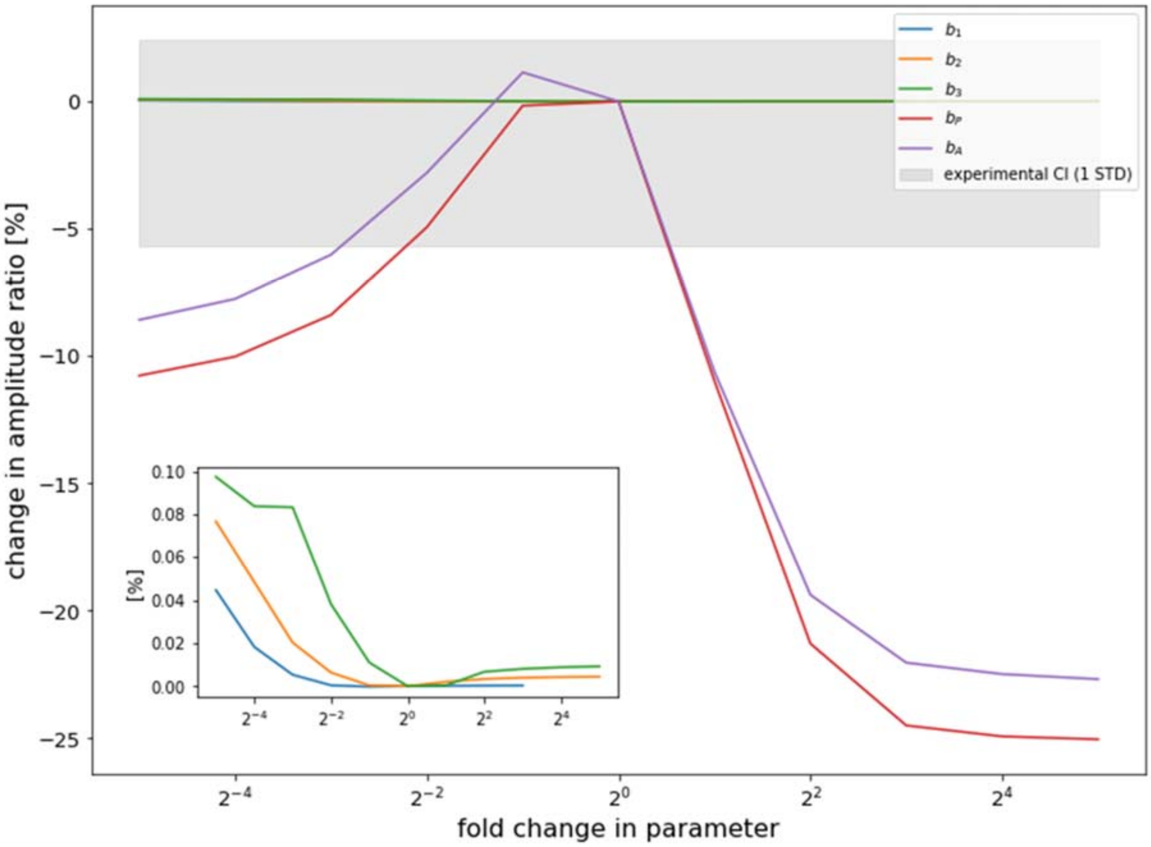

Figure S3. Sensitivity analysis of HPA model with mass dynamics suggests that the dominance of the low frequency is insensitive to all model parameter, related to Figure 4 and Table S1. Each of the five model parameters was varied around its reference range by a range of $2^{10} = 1024$. For each case, the simulation of Figure 4 was repeated, and the ratio between the $1\ year^{-1}$ and $3\ year^{-1}$ amplitudes was computed. The plot shows the percent change in the ratio as a function of the fold change in each parameter. The slow-timescale parameters for the tissue turnover

processes, b_A and b_P, match the observe ratio within its experimental error (gray region) for an approximately 8-fold range. The fast timescale parameters of hormone production and removal have minor effects on the ratio across the entire range (inset).

S6. Frequency response of the linearized HPA model

In order to put the findings in perspective, and to add analytical understanding, we calculate here the frequency response of the linearized HPA model. The frequency response allows one to calculate the magnitude and the phase of the system's output as a function of frequency, for a given input. We analyze the system around its steady state. Although in reality the simulations are not strictly in the linear range, the linear approximation can be used to gain intuition. We thus assume $x_3 << K_{GR}$, therefore the GR is not activated and we can set $GR(x) = 1$. Substituting this in the model equations (1)-(7) (Methods). We used dimensionless variables for the hormones and glands. We do so by normalizing the steady-state of all variables to be 1 for an input $u = 1$. Scaling the variables gives:

$$\frac{dx_1}{dt} = b_1\left(\frac{u}{x_3} - x_1\right)$$

$$\frac{dx_2}{dt} = b_2(Cx_1 - x_2)$$

$$\frac{dx_3}{dt} = b_3(Ax_2 - x_3)$$

$$\frac{dC}{dt} = b_C C(x_1 - 1)$$

$$\frac{dA}{dt} = b_A A(x_2 - 1)$$

The linearization of these equations around their steady state and using a Laplace transform gives:

$$sX_1(s) = b_1\big(U(s) - X_1(s) - X_3(s)\big)$$

$$sX_2(s) = b_2\big(X_1(s) - X_2(s) + C(s)\big)$$

$$sX_3(s) = b_3\big(X_2(s) - X_3(s) + A(s)\big)$$

$$sC(s) = b_C X_1(s)$$

$$sA(s) = b_A X_2(s)$$

The capital letters denote the Laplace transform of each variable. Solving these equations we get:

$$H_1(s) = \frac{X_1(s)}{U(s)} = \frac{b_1(b_2 + s)(b_3 + s)s^2}{D(s)}$$

$$H_2(s) = \frac{X_2(s)}{U(s)} = \frac{b_1b_2(b_3 + s)(b_C + s)s}{D(s)}$$

$$H_3(s) = \frac{X_3(s)}{U(s)} = \frac{b_1b_2b_3(b_C + s)(b_A + s)}{D(s)}$$

$$H_C(s) = \frac{C(s)}{U(s)} = \frac{b_1b_C(b_2 + s)(b_3 + s)s}{D(s)}$$

$$H_A(s) = \frac{A(s)}{U(s)} = \frac{b_1b_2b_A(b_3 + s)(b_C + s)}{D(s)}$$

Where $D(s) = s^5 + (b_1 + b_2 + b_3)s^4 + (b_1b_2 + b_1b_3 + b_2b_3)s^3 + 2b_1b_2b_3s^2 + b_1b_2b_3(b_A + b_C)s + b_1b_2b_3b_Ab_C$

$H_x(s)$ are the transfer functions of each variable. By substituting $s = jw$ ($j = \sqrt{-1}$), we obtain the frequency response of the system. The amplitude of each variable as function of frequency is obtained by calculating the magnitude of the transfer function, $A_x = |H_x(jw)|$. Using the parameters listed in table S1 we plot the cortisol frequency response (Figure S4). Note that a white noise input corresponds to input with constant amplitude at each frequency. Thus, the frequency response curve corresponds to the Fourier amplitudes expected for a white noise input. At very high frequencies corresponding to periods of days or hours, the response decays. At frequencies of around $1\,year^{-1}$ the frequency response shows the qualitative trend observed in the present study: the $1\,year^{-1}$ frequency has higher amplitude than the $2\,year^{-1}$ and $3\,year^{-1}$ frequencies as found in the results section. Note that frequencies slower than $1\,year^{-1}$ are also predicted to occur. Observing such variations with a period longer than a year require a longer longitudinal study.

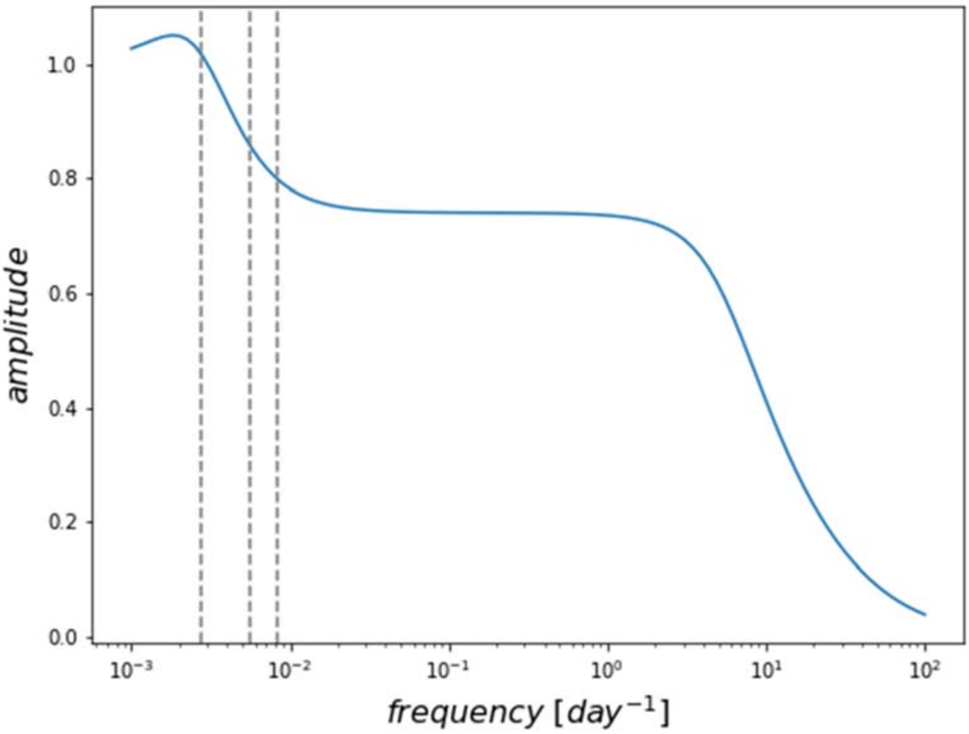

Figure S4. Analytical linearized frequency response for cortisol shows dominant $year^{-1}$ frequency, related to Figure 3. Cortisol amplitude as function of the stressor input frequency in the HPA model with mass dynamics. Vertical dashed lines correspond to the frequencies in the hair cortisol experiment - $1,2\ and\ 3\ year^{-1}$.

S7. Analysis of the gland turnover times

Combining theoretical hypotheses and experimental evidence, we construct different independent constraints on the relationships between the parameters of the gland turnover rates (a_C and a_A). Each constraint is projected onto the parameter space and is satisfied in some region of this space (Figure S5). The intersection (if it exists) between these different regions provides a range of consistent parameter values.

Using the linearized frequency response of the HPA system (SI, S6) and the experimental results of this study, one can constrain the turnover parameters to fulfill a condition on the ratio between the cortisol amplitudes $|H_3|$ at the slowest frequency ($1\ year^{-1}$), to the fastest ($3\ year^{-1}$). The experimentally observed ratio is in the range

$$1.3 < \frac{|H_3(w = 1\ year^{-1})|}{|H_3(w = 3\ year^{-1})|} < 1.65$$

We added this constraint to the ones discovered in a previous study (Tendler et al., 2020) on seasonal entrainment of the hormone circuit: 1) A resonance frequency of approximately 1 year (range 9-13 months) for the gland-mass negative feedback circuit; 2) Experimental observation that cortisol blood and urine tests peak at late winter.

These constraints are independent, and hence a-priori they do not have to converge in a specific intersection region. The existence of a region (green region) provides a consistent parameter range. Reassuringly, this range is consistent with gland turnover measurements in model organisms (GERTZ et al., 1987; Kataoka et al., 1996; Nolan et al., 1998; WESTLUND et al., 1985).

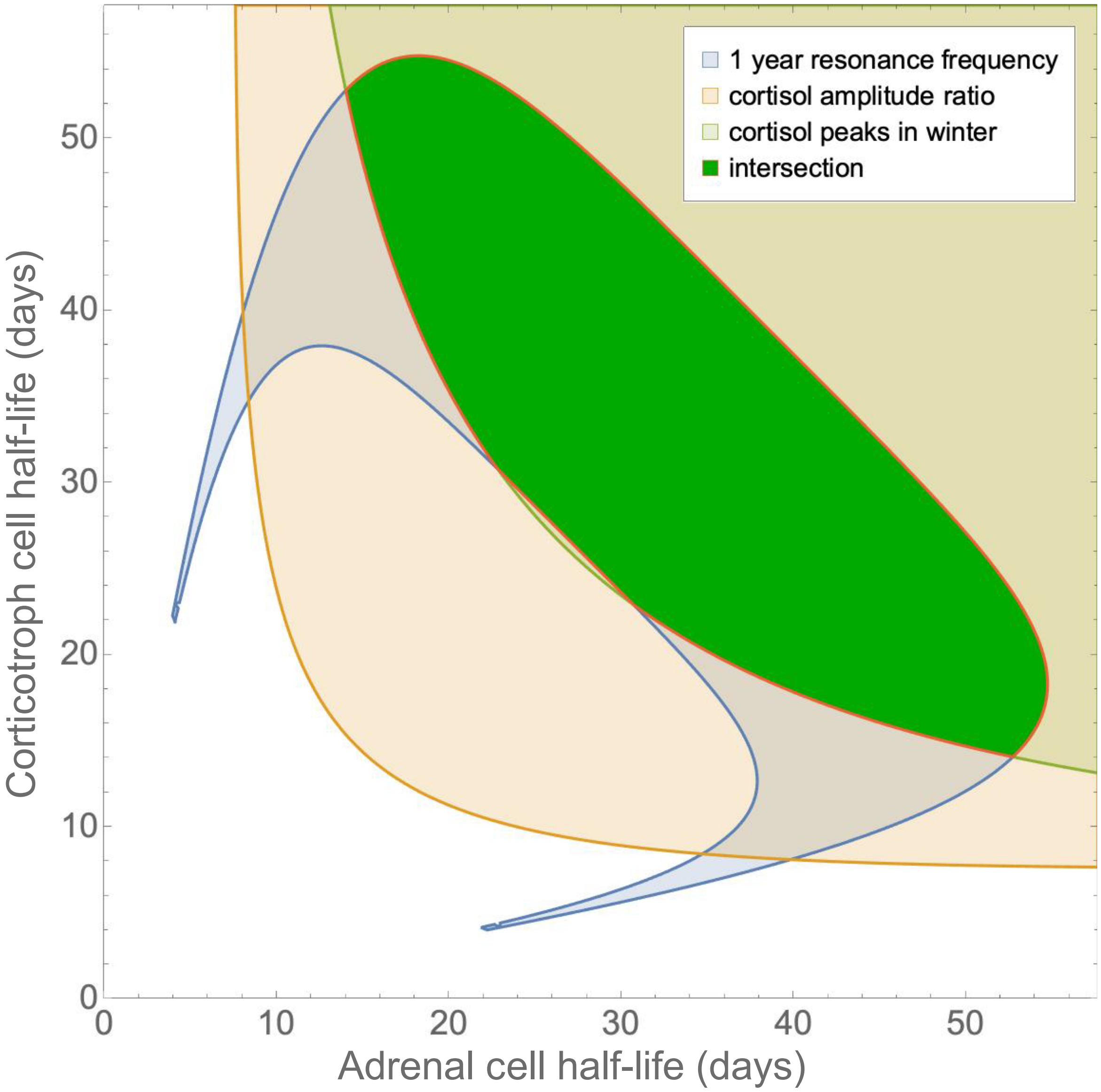

Figure S5. Constraint analysis for gland turnover parameters suggests a range consistent between several independent measurements, related to Figure 4 and Table S1. Each region corresponds to parameter values that satisfy a certain constraint. (i) ratio between $1\ year^{-1}$ and $3\ year^{-1}$ frequencies in response to white noise in the present experimental range (orange region). (ii) cortisol peak in winter for a seasonal input as measured and calculated in Tendler et al. (light green) (iii) resonance frequency of the linearized model in the range of 9-13 months (blue region). Intersection: dark green region.
